# Assessment of Technology Readiness in Norwegian Older Adults With Long-Term Health Conditions Receiving Home Care Services: Cross-Sectional Questionnaire Study

**DOI:** 10.2196/62936

**Published:** 2025-02-07

**Authors:** Sverre Bergh, Jūratė Šaltytė Benth, Lisbeth Dyrendal Høgset, Britt Rydjord, Lars Kayser

**Affiliations:** 1 Research Centre for Age-related Functional Decline and Disease Innlandet Hospital Trust Ottestad Norway; 2 Norwegian National Centre for Aging and Health Vestfold Hospital Trust Tønsberg Norway; 3 Institute for Clinical Medicine University of Oslo Oslo Norway; 4 Health Services Research Unit Akershus University Hospital Oslo Norway; 5 Department for Research and Innovation Innlandet Hospital Trust Ottestad Norway; 6 Department of Public Health University of Copenhagen Copenhagen Denmark

**Keywords:** eHealth literacy, digital health services, technology readiness, Readiness and Enablement Index for Health Technology, READHY, chronic conditions

## Abstract

**Background:**

With the increasing number of older adults globally, there is a constant search for new ways to organize health care services. Digital health services are promising and may reduce workload and at the same time improve patient well-being. A certain level of eHealth literacy is needed to be able to use digital health services. However, knowledge of technology readiness in this target group of older adults is unclear.

**Objective:**

The aim of this study was to understand the technology readiness level of a group of older adults who were provided home care services in order to address the present and future needs of this group in relation to the implementation of digital health care services.

**Methods:**

This quantitative cross-sectional study included 149 older adults from Norway receiving home care services. The participants completed the Readiness and Enablement Index for Health Technology (READHY) instrument, assessments of well-being (World Health Organization-Five Well-Being Index [WHO-5]), and assessments of demographic and clinical variables (sex, age, education, living situation, comorbidity, use of digital devices, and use of IT). Cluster analyses were used to group the users according to their technology readiness.

**Results:**

The mean participant age was 78.6 (SD 8.0) years, and 55.7% (83/149) were women. There was good consistency within the assumed READHY scales (Cronbach α=.61-.91). The participants were grouped into 4 clusters, which differed in terms of READHY scores, demographic variables, and the use of IT in daily life. Participants in cluster 1 (n=40) had the highest scores on the READHY scales, were younger, had a larger proportion of men, had higher education, and had better access to digital devices and IT. Participants in cluster 4 (n=16) scored the lowest on eHealth literacy knowledge. Participants in cluster 1 had relatively high levels of eHealth literacy knowledge and were expected to benefit from digital health services, while participants in cluster 4 had the lowest level of eHealth literacy and would not easily be able to start using digital health services.

**Conclusions:**

The technology readiness level varied in our cohort of Norwegian participants receiving home care. Not all elderly people have the eHealth literacy to fully benefit from digital health services. Participants in cluster 4 (n=16) had the lowest scores in the eHealth Literacy Questionnaire scales in the READHY instrument and should be offered nondigital services or would need extensive management support. The demographic differences between the 4 clusters may inform stakeholders about which older people need the most training and support to take advantage of digital health care services.

## Introduction

In the coming years, the world will have an increasing number of older adults. According to the World Health Organization (WHO), 1 in 6 people in the world will be 60 years or older in 2030 [1]. Today’s aging population is more healthy than previous generations, with a longer life expectancy and more years without frailty [2]. Nevertheless, in the future, there will be more older adults with long-term health conditions and frailty [1]. This will further increase the burden on health care services in the coming years. This change in demographics together with a reduced workforce due to a reduction in the birth rate calls for new ways to organize health and care services. There is an urgent need for more efficient ways to provide the needed health care with fewer in-person contacts, the involvement of informal caregivers, an increase in self-management, and a reduction in travel time, particularly in rural areas. Increased digitalization of health care services could be a solution, which could enable and engage more patients, resulting in increased levels of self-efficacy, self-management, and empowerment [3], and thus reduce the burden on the workforce [4]. Moreover, telehealth solutions may increase the number of virtual contacts and provide means for self-monitoring and dialogues with health professionals as an immediate response to the early signs of deterioration in the health condition [5]. The use of telemedicine may be of particular importance in rural areas with scattered settlements, saving travel time and costs for both health personnel and patients. Although evidence for the assumed benefits of the digital transformation of health care services is scarce, particularly among older adults, it will continue in the coming years along with the general digitalization of both private and public services in Norway and many other countries.

In 2021, 96% of Norwegians older than 9 years had access to a smartphone [6]. However, this indicates that 4%, or approximately 200,000 people, do not have a smartphone. A higher number of people probably have an insufficient level of digital health literacy. In a study from 2021, the Norwegian Directorate of Health found that 13% of people aged ≥60 years never used the internet, while 9% used the internet only once a week. Moreover, only 66% of people aged ≥80 years used the internet. Further, 44% of people aged ≥60 years reported no or low experience with digital IDs and 2-factor identification, which are needed to access public web pages [7].

One benefit from the digital health transformation is that citizens and patients may communicate from their homes and receive care and treatment virtually. Services may also be provided by specialists at hospitals in collaboration with registered nurses in municipalities and the person’s own general practitioner, offering better coordination. This requires the individuals being cared for to have access to the internet; have devices, such as smartphones, tablets, laptops, and PCs; and have the ability to use these devices. For those not having these resources, it is essential to identify this challenge and plan for the involvement of supporting resources or nondigital services. This calls for new ways to plan for digitally enabled services and usage of digital tools, and to be aware of the specific resources and needs of individuals when offering these.

In this context, awareness of the person’s level of digital health literacy [8] and level of technology readiness [9] is important and may be helpful information together with sociodemographic characteristics, including access to and familiarity with digital devices. In particular, older adults have a lower level of digital health literacy, and as lower levels of digital health literacy may be related to economic status, it may be hypothesized that older people living in rural areas with precisely these characteristics may have a low or even insufficient level of digital health literacy. Technology readiness may be a barrier for offering telehealth solutions, which otherwise would be of huge benefit to these people, due to the fact that the remote areas of living may prohibit access to in-person services, particularly outside the summer season. This calls for knowledge about the levels of digital health literacy and technology readiness of different populations.

eHealth literacy, which is currently often used synonymously with digital health literacy, was defined in 2006 by Norman and Skinner as “the ability to seek, find, understand, and appraise health information from electronic sources and apply the knowledge gained to addressing or solving a health problem” [8]. Several factors, such as frailty, low level of digital health literacy, and motoric or cognitive impairment, may be barriers for patients when introducing new technologies [10].

One way to identify barriers is by measuring the level of digital health literacy using a multi-faceted instrument such as the eHealth Literacy Questionnaire (eHLQ) [11]. Another way is to expand on the factors necessary for being able to apply digital health literacy in a context, by using the Readiness and Enablement Index for Health Technology (READHY) [9]. The READHY instrument builds on the eHLQ but includes 4 scales related to self-management from the Health Education Impact Questionnaire (heiQ) [12] and 2 scales related to support from the Health Literacy Questionnaire (HLQ) [13]. The READHY instrument has been proven to inform about variations in needs and skills in relation to technology readiness presented as specific clusters.

The READHY instrument can be used both as a framework for qualitative studies [14] and as an instrument to assess the experience of support, elements of self-management, and levels of digital health literacy. It has been used in a number of contexts, such as cancer survivors [15], type 2 diabetes mellitus (T2DM) patients [16], older hospitalized medical patients, and people living with inflammatory bowel disease [17,18]. The separate scales of the READHY instrument have been translated and validated in the Norwegian language, but the complete READHY instrument has not yet been published in Norwegian research [19,20].

Despite emerging knowledge within various specific conditions, studies focusing on the technology readiness of older adults are lacking. People receiving home care services may have other needs for digital health services than people with specific diseases, such as cancer and T2DM, and it is important to have an instrument that is broad enough to catch these differences. In our study, the READHY instrument will be tested as an option to elucidate the characteristics of this population of older adults living in a rural area.

Data obtained using the READHY instrument can be clustered to identify segments of users, and this segmentation can be enriched with other characteristics, such as sociodemographics, health conditions, and well-being, forming clusters to help health professionals recognize the need for support or training among the people they serve [21]. This information may help health service organizations to plan for subpopulations based on the identified clusters of the general population and with knowledge about the size of particular groups. It may also help health care professionals to have better conversations when introducing new digital health tools or offering telehealth services to ensure meaning and motivation for older adults [22].

The aim of this study was to explore the technology readiness level in a population of older adults living in a rural area, a group that is hypothesized to be less technology ready. Further, the study attempted to examine their digital resources and behaviors and evaluate associations between these factors and their self-reported health and mental well-being, which may influence their motivation and ability to create meaning in using digital health services and tools.

The results of the study may help service providers to better plan for the specific needs of subgroups and provide health care professionals with insights that may help them to better understand how to introduce new digital health tools and services in this group of older adults living in rural areas. This group is more likely to be frail and at the same time may benefit more from virtual and other contacts that are not in-person. Using clustering analysis, we explored the technology readiness level of older adults receiving home care and their characteristics in terms of resources and digital behaviors (ie, sociodemographic variables and use of digital devices). 

## Methods

### Design

This cross-sectional study included 149 older adults recruited from 7 municipalities in South-Eastern Norway. The data were collected by 1 to 2 health care staff in each of the 7 participating municipalities. The same research nurse trained all data collectors (2-hour training session), and the data collectors were supervised by the research nurse during the study period. The participants completed the questionnaires listed below, either self-reported by themselves or in an interview with the health care staff. The participants were approached in a face-to-face setting where they were informed about the study, signed the consent form, and completed the questionnaires.

### Population

Health care staff in the municipal home care services and at the lung department of Innlandet Hospital Trust recruited the participants. Patients admitted to the outpatient clinic at the lung department and people receiving home care services from the municipalities were invited to participate in the study. The inclusion criteria were as follows: (1) living at home, (2) ≥65 years of age, (3) receiving home care services, (4) presence of long-term health conditions, (5) fluency in Norwegian, and (6) written informed consent. The exclusion criteria were as follows: (1) severe dementia (Clinical Dementia Rating scale score of 3), (2) terminal disease with <3 months of expected survival, and (3) severe psychiatric disease, including drug abuse. A total of 211 individuals were eligible, of which 62 were not included as they did not consent to participate. Those not included were on average 83.5 (SD 5.7) years old, and 61.3% were female. The process of participant inclusion was from September 2021 to June 2023.

### Included Data and Questionnaires

We collected data on age (years), sex (male/female), living situation (alone, with spouse/partner, or with others), comorbidity, education (compulsory lower secondary school, upper secondary education, master’s degree, or more than 4 years university), use of digital devices (laptop, PC, tablet, or smartphone), use of technology to access the general physician and electronic medical record, and digital competence (very poor, poor, average, good, or very good).

The READHY instrument consists of 65 items, with 4 scales from the heiQ [12], 2 scales from the HLQ [13], and all scales from the eHLQ (Table 1) [11]. The heiQ is used to assess patient education and self-management and is validated in Norwegian [12,19]. The HLQ is used to assess health literacy and has been translated to and validated in many languages including Norwegian [13,23]. The eHLQ is used to assess the benefits of telemedicine for individuals, evaluate how telehealth services work together, and examine the telemedicine knowledge of individuals [18]. It has been translated, validated, and psychometrically tested in Norwegian [24]. The full READHY instrument is validated in several languages but not yet in Norwegian. The READHY instrument assesses the person’s health technology readiness [9]. Each item in the READHY instrument can be scored from 1 to 4 (strongly disagree, disagree, agree, and strongly agree). The scoring of “strongly agree” in the item heiQ8 *emotional distress* is reversed for the statistical analysis, with the highest score representing the lowest level of distress. The scores of the scales in the READHY instrument are calculated as the mean scores of the included items for each scale. The scores of scales with missing data have been included if the number of missing items is ≤50%.

**Table 1:** Scales from the Health Education Impact Questionnaire (heiQ), Health Literacy Questionnaire (HLQ), and eHealth Literacy Questionnaire (eHLQ) included in the Readiness and Enablement Index for Health Technology (READHY).

Questionnaire and scale		Description
**heiQ^a^**		
	heiQ3	Self-monitoring and insight
	heiQ4	Constructive attitudes and approaches
	heiQ5	Skill and technique acquisition
	heiQ8	Emotional distress
**HLQ^b^**		
	HLQ1	Feeling understood and supported by health care providers
	HLQ4	Social support for health
**eHLQ^c^**		
	eHLQ1	Using technology to process health information
	eHLQ2	Understanding of health concepts and language
	eHLQ3	Ability to actively engage with digital services
	eHLQ4	Feeling safe and in control
	eHLQ5	Motivated to engage with digital services
	eHLQ6	Access to digital services that work
	eHLQ7	Digital services that suit individual needs

^a^heiQ: Health Education Impact Questionnaire.

^b^HLQ: Health Literacy Questionnaire.

^c^eHLQ: eHealth Literacy Questionnaire.

Cutoff values are not meaningful from a psychometric approach, and the values for scoring in the READHY scales have not been calibrated to each other or to outcomes. Despite this, scores below 2.7 on the scales may mirror that the person has a level of “not being sufficient” as not all items in the scales are scored as “strongly agree” or “agree.” A score of 2.0 or lower on the scales may be considered problematic for managing digital health services as most items in the scales are scored as “strongly disagree” or “disagree” [25]. The READHY instrument takes 10 to 20 minutes to complete in most populations (including older adults), but it could take up to 30 minutes to complete for individuals with cognitive impairment.

The World Health Organization-Five Well-Being Index (WHO-5) consists of 5 items. Each item is scored from 0 to 5, where 5 indicates *all the time* and 0 indicates *at no time*. The score of each item is multiplied by 4, giving a total score from 0 to 100.

Some additional questions were asked to enrich the data. A question about digital competence was asked: “If someone knowing you well should assess your IT competence, would they say it is very poor/poor/average/good/very good?” A question about the participants’ health was taken from the 36-item Short-Form Survey instrument (SF-36): “In general, would you say your health is excellent/very good/good/fair/poor?” Scores range from 1 (excellent) to 5 (poor) [26], and they were treated as a continuous variable.

### Statistical Analysis

The participant characteristics have been presented as means and SDs or frequencies and percentages, as appropriate. The READHY scale score was calculated as the mean of the item scores when fewer than half of the items had missing values. This resulted in 2 excluded cases where the scale score could not be calculated. Ideally, one should start with assessing the dimensionality of the READHY instrument by applying a factor analysis. However, due to convergence problems caused by a very small sample size with respect to the number of items, this approach was not possible. We therefore report the Cronbach α for the scales identified by a previous factor analysis of a Danish dataset [9].

To identify groups of participants based on the READHY scales, we first applied a hierarchical cluster analysis with Euclidean squared distance and Ward linkage to identify the possible number of groups [27]. The results of the hierarchical cluster analysis were then used to determine an appropriate number of clusters in the next step, a k-means cluster analysis [28] with Euclidean similarity measure. By examining the dendrogram from the first step, we expected to gain insights into the natural grouping of data, which would inform the subsequent k-means analysis. In this way, we leveraged the strengths of both methods for more effective clustering and based our conclusion on k-means analysis, which is easier to interpret due to each individual being assigned to one cluster. While choosing the final number of clusters, we considered clinical relevance, cluster size, small within-cluster variability, and between-cluster heterogeneity.

One-way ANOVA was then applied to assess the differences between the identified clusters with respect to the READHY scales. Further, one-way ANOVA, the chi-square test, and the Fisher exact test were applied to compare the profiles of the identified clusters based on the following variables: all scale scores, age, sex, education (≤12 years or >12 years), living situation (alone, with spouse/partner, or with others), comorbidity (number of chronic diagnoses), use of a laptop, PC, or tablet at least once a week, use of a smartphone at least once a week, use of IT in previous work or studies, use of IT to communicate with public services, use of the Norwegian digital identifier to access a webpage, use of IT to communicate with a general practitioner, logging into a national health webpage, use of IT to find health information on the internet or social media, reading medical journals or test results on a national health webpage, IT competence assessed by others (very poor, poor, average, good, or very good), WHO-5 score, and response to the question “In general, would you say your health is excellent/very good/good/fair/poor?” Pairwise comparisons of groups were performed as post hoc analyses. The results of these post hoc analyses were reported as mean differences and SDs for continuous variables, mean differences and SDs in proportions for dichotomous variables, and effect sizes calculated as absolute values of z-statistics from the Mann-Whitney *U* test divided by the square root of the total sample size for ordinal variables, along with *P* values. Only significant pairwise differences were tabulated.

Two linear regression models were estimated to assess the associations of WHO-5 (primary) and IT competence assessed by others (secondary) with prechosen covariates: age, sex, education (≤12 years or >12 years), living situation (alone, with spouse/partner, or with others), comorbidity, use of a laptop, PC, or tablet at least once a week, use of a smartphone at least once a week, use of IT to communicate with a general practitioner, use of IT to find health information on the internet or social media, reading medical journals or test results on a national health webpage, and response to the question “In general, would you say your health is excellent/very good/good/fair/poor?” The regression models were estimated in cases with no missing values for covariates.

Statistical analyses were performed using STATA version 18 (StataCorp). A *P* value of <.05 was considered statistically significant. No adjustment for multiple testing was applied; however, we separated between ANOVA as the main analysis and post hoc analyses considered purely exploratory. Although we have only reported the results of the post hoc analyses with *P*<.05, we emphasize that all pairwise comparisons were performed. Further, the regression analyses have been considered secondary. Moreover, there is clarity regarding the number of tests performed, allowing the reader to consider the reliability of our findings.

### Ethical Considerations

Participation in the study was based on informed written consent. The study was considered by The Regional Committee for Medical and Health Research Ethics in Norway as being outside its jurisdiction. The data collection, data storage, data analysis, and publication of the results were approved by the data protection officer at Innlandet Hospital Trust (number: 14832226). Participants could withdraw from the study at any time and request for their data to be deleted.

## Results

The descriptive results of the cohort are presented in Table 2. The mean age of the participants was 78.6 (SD 8.0) years, and 55.7% (83/149) were women. Moreover, 19.2% (27/141) had higher education (>12 years) and 69.8% (104/149) were living alone. The mean scores of the READHY scales ranged from 2.0 (SD 0.5) for the eHLQ1 to 3.1 (SD 0.3) for the heiQ3. The heiQ scales assessed patient education and self-management, and the mean scores for the 4 scales ranged from 2.8 (SD 0.6) to 3.1 (SD 0.3), indicating high levels of patient education and self-management. The HLQ scales assessed health literacy, and the mean scores ranged from 2.9 (SD 0.4) to 3.0 (SD 0.5), indicating a high level of health literacy. The eHLQ scales assessed eHealth literacy, and the mean scores ranged from 2.0 (SD 0.5) to 3.0 (SD 0.3), indicating that eHealth literacy was low in our population. Moreover, the scores differed between the scales. Table 3 presents Cronbach α values for the READHY scales. Except for the eHLQ2 scale with a low Cronbach α of .61, the Cronbach α values ranged from .70 to .91 for the READHY scales, showing good consistency within the assumed scales [29].

**Table 2:** Summarized statistics of the cohort.

Variable				Value (N=149)
Age (years) (n=148), mean (SD)				78.6 (8.0)
Gender (female), n (%)				83 (55.7)
**Education (n=141), n (%)**				
	≤12 years			114 (80.9)
	>12 years			27 (19.2)
**Living situation, n (%)**				
	Living alone			104 (69.8)
	Living with spouse/partner/others			45 (30.2)
Number of chronic diagnoses (n=144), mean (SD)				2.6 (1.4)
**Use of digital devices at least once a week, n (%)**				
	PC, laptop, or tablet			72 (48.3)
	Smartphone			89 (59.7)
	IT to communicate with a general practitioner			34 (22.8)
	IT to find health information on the internet or social media			17 (11.6)
**Reading medical journals or test results on a national health webpage, n (%)**				
	Never/rarely			116 (78.4)
	Sometimes/always			32 (21.6)
In general, would you say your health is excellent/very good/good/fair/poor, mean (SD)				1.2 (0.8)
**READHY^a^ scales, mean (SD)**				
	**heiQ^b^**			
		heiQ3	3.1 (0.3)	
		heiQ4	2.9 (0.4)	
		heiQ5	2.8 (0.4)	
		heiQ8	2.8 (0.6)	
	**HLQ^c^**			
		HLQ1 (n=148)	3.0 (0.5)	
		HLQ4 (n=148)	2.9 (0.4)	
	**eHLQ^d^**			
		eHLQ1 (n=147)	2.0 (0.5)	
		eHLQ2	2.8 (0.4)	
		eHLQ3 (n=147)	2.2 (0.6)	
		eHLQ4	3.0 (0.3)	
		eHLQ5	2.2 (0.5)	
		eHLQ6	2.4 (0.4)	
		eHLQ7 (n=148)	2.2 (0.6)	

^a^READHY: Readiness and Enablement Index for Health Technology.

^b^heiQ: Health Education Impact Questionnaire.

^c^HLQ: Health Literacy Questionnaire.

^d^eHLQ: eHealth Literacy Questionnaire.

**Table 3:** Cronbach α values of the Readiness and Enablement Index for Health Technology (READHY) scales.

Scale		Cronbach α
**heiQ^a^**		
	heiQ3: Self-monitoring and insight (6 items)	.69
	heiQ4: Constructive attitudes and approaches (5 items)	.77
	heiQ5: Skills and technique acquisition (4 items)	.70
	heiQ8: Emotional distress (6 items)	.90
**HLQ ^b^**		
	HLQ1: Feeling understood and supported by health care providers (4 items)	.83
	HLQ4: Social support for health (5 items)	.77
**eHLQ^c^**		
	eHLQ1: Using technology to process health information (5 items)	.86
	eHLQ2: Understanding of health concepts and language (5 items)	.61
	eHLQ3: Ability to actively engage with digital services (5 items)	.91
	eHLQ4: Feeling safe and in control (5 items)	.71
	eHLQ5: Motivated to engage with digital services (5 items)	.80
	eHLQ6: Access to digital services that work (6 items)	.78
	eHLQ7: Digital services that suit individual needs (4 items)	.85

^a^heiQ: Health Education Impact Questionnaire.

^b^HLQ: Health Literacy Questionnaire.

^c^eHLQ: eHealth Literacy Questionnaire.

Based on visually inspecting a dendrogram from the hierarchical cluster analysis (not presented), exploring 3 to 6 cluster solutions by k-means cluster analysis, and considering clinical relevance, within-cluster variability, between-cluster heterogeneity, and reasonable sample size, the 4-cluster model was chosen (Table 4). The details of patient profiles in the 4 clusters are described in Multimedia Appendix 1. The participants in cluster 1 (n=40) had on average the highest scores on the READHY scales (meaning better eHealth literacy), except the HLQ4 scale. The HLQ4 scale assesses the social support for health, where the scores were intermediate. Participants in cluster 1 were younger than the other participants (*P*=.006), had a larger proportion of men (*P*<.001), had higher education (*P*<.001), had better access to smartphones and PCs, laptops, or tablets (*P*<.001), had used IT more in their previous work (*P*<.001), and currently used IT to access health information and stay in touch with the health care system (*P*<.001). The participants in cluster 3 (n=25) had the lowest average scores on the heiQ4, heiQ5, and heiQ8 scales (patient education and self-management) and the 2 HLQ scales (feeling understood and supported by health care providers, and social support for health). The participants in cluster 4 (n=16) had the lowest average scores on the eHLQ scales (meaning low eHealth literacy knowledge), except the eHLQ4 scale (feeling safe and in control), where their scores were comparable to those of the 3 other clusters. Thus, the feelings of being safe and in control were equal in the 4 clusters. Cluster 2 was the largest (n=66) and had intermediate scores on most of the scales. Interestingly, the question “IT competence assessed by others” differentiated between all clusters, except between cluster 2 and cluster 3 (*P*<.001).

**Table 4:** Results of k-means clustering with 4 clusters (n=147).

Scale			Cluster 1 (n=40), mean (SD)		Cluster 2 (n=66), mean (SD)		Cluster 3 (n=25), mean (SD)		Cluster 4 (n=16), mean (SD)		*P* value for ANOVA		*P* value for pairwise comparisons, mean difference (SD) and *P* value
**heiQ^a^**													
	heiQ3	3.2 (0.3)		2.9 (0.3)		3.0 (0.3)		3.2 (0.5)		<.001		1 vs 2: 0.3 (0.1), *P*<.001; 1 vs 3: 0.2 (0.1), *P*=.01; 2 vs 4: –0.2 (0.1), *P*=.009	
	heiQ4	3.1 (0.3)		3.0 (0.3)		2.4 (0.3)		3.0 (0.5)		<.001		1 vs 3: 0.7 (0.1), *P*<.001; 2 vs 3: 0.6 (0.1), *P*<.001; 3 vs 4: –0.6 (0.1), *P*<.001	
	heiQ5	2.9 (0.4)		2.8 (0.3)		2.6 (0.5)		3.0 (0.5)		.001		1 vs 2: 0.1 (0.1), *P*=.047; 1 vs 3: 0.4 (0.1), *P*=.001; 2 vs 3: 0.3 (0.1), *P*=.004; 3 vs 4: –0.4 (0.2), *P*=.01	
	heiQ8	2.9 (0.6)		3.0 (0.3)		2.1 (0.5)		2.8 (0.7)		<.001		1 vs 3: 0.8 (0.1), *P*<.001; 2 vs 3: 0.9 (0.1), *P*<.001; 3 vs 4: –0.7 (0.2), *P*=.002	
**HLQ^b^**													
	HLQ1	3.2 (0.5)		2.9 (0.4)		2.9 (0.3)		3.0 (0.7)		.003		1 vs 2: 0.3 (0.1), *P*<.001; 1 vs 3: 0.3 (0.1), *P*=.006	
	HLQ4	3.0 (0.4)		2.8 (0.4)		2.6 (0.4)		3.2 (0.4)		<.001		1 vs 3: 0.3 (0.1), *P*=.004; 2 vs 3: 0.2 (0.1), *P*=.009; 2 vs 4: –0.4 (0.1), *P*=.001; 3 vs 4: –0.6 (0.1), *P*<.001	
**eHLQ^c^**													
	eHLQ1	2.5 (0.5)		2.0 (0.2)		2.1 (0.3)		1.1 (0.2)		<.001		1 vs 2: 0.6 (0.1), *P*<.001; 1 vs 3: 0.4 (0.1), *P*=.001; 1 vs 4: 1.4 (0.1), *P*<.001; 2 vs 3: –0.2 (0.1), *P*=.002; 2 vs 4: 0.8 (0.1), *P*<.001; 3 vs 4: 1.0 (0.1), *P*<.001	
	eHLQ2	3.1 (0.3)		2.6 (0.3)		2.8 (0.2)		2.6 (0.5)		<.001		1 vs 2: 0.4 (0.1), *P*<.001; 1 vs 3: 0.3 (0.1), *P*<.001; 1 vs 4: 0.4 (0.1), *P*<.001; 2 vs 3: –0.2 (0.1), *P*=.02	
	eHLQ3	2.9 (0.5)		2.1 (0.3)		2.3 (0.4)		1.1 (0.2)		<.001		1 vs 2: 0.8 (0.1), *P*<.001; 1 vs 3: 0.6 (0.1), *P*<.001; 1 vs 4: 1.8 (0.1), *P*<.001; 2 vs 3: –0.2 (0.1), *P*=.004; 2 vs 4: 1.0 (0.1), *P*<.001; 3 vs 4: 1.2 (0.1), *P*<.001	
	eHLQ4	3.1 (0.4)		3.0 (0.2)		3.0 (0.2)		3.1 (0.4)		.08		1 vs 2: 0.1 (0.1), *P*=.02	
	eHLQ5	2.7 (0.5)		2.0 (0.2)		2.1 (0.3)		1.5 (0.4)		<.001		1 vs 2: 0.6 (0.1), *P*<.001; 1 vs 3: 0.6 (0.1), *P*<.001; 1 vs 4: 1.2 (0.1), *P*<.001; 2 vs 4: 0.5 (0.1), *P*<.001; 3 vs 4: 0.6 (0.1), *P*<.001	
	eHLQ6	2.9 (0.3)		2.3 (0.2)		2.3 (0.3)		1.6 (0.3)		<.001		1 vs 2: 0.6 (0.05), *P*<.001; 1 vs 3: 0.5 (0.1), *P*<.001; 1 vs 4: 1.2 (0.1), *P*<.001; 2 vs 4: 0.6 (0.1), *P*<.001; 3 vs 4: 0.7 (0.1), *P*<.001	
	eHLQ7	2.8 (0.4)		2.0 (0.2)		2.0 (0.3)		1.2 (0.4)		<.001		1 vs 2: 0.8 (0.1), *P*<.001; 1 vs 3: 0.8 (0.1), *P*<.001; 1 vs 4: 1.6 (0.1), *P*<.001; 2 vs 4: 0.8 (0.1), *P*<.001; 3 vs 4: 0.8 (0.1), *P*<.001	

^a^heiQ: Health Education Impact Questionnaire.

^b^HLQ: Health Literacy Questionnaire.

^c^eHLQ: eHealth Literacy Questionnaire.

In the bivariate linear regression model, WHO-5 as an outcome (Multimedia Appendix 2) was significantly associated with education and the question “In general, would you say your health is excellent/very good/good/fair/poor?”, while only the latter question remained significant in the multiple model. A 1-point increase in the score of the question “In general, would you say your health is excellent/very good/good/fair/poor?” was associated with a 12.7-point increase in the WHO-5 score.

In the multiple linear regression model with the question “IT competence assessed by others” as an outcome (Multimedia Appendix 3), only “use of a PC, laptop, or tablet at least once in the last week” and “reading medical journals or test results on a national health webpage” were significantly associated with the outcome.

## Discussion

### Summary of the Main Findings

In this study, we included participants from a rural area in Norway with less education than other parts of Norway, and our recruitment procedure resulted in the inclusion of slightly more women than men. The participants were assessed for technology readiness using the READHY instrument. We found that people receiving home care services in this area had high levels of patient education and self-management (scores of 2.8-3.1) and high levels of health literacy (scores of 2.9-3.0) but generally low levels of eHealth literacy (scores of 2.0-3.0). The findings were based on the assumption that a score of ≥2.7 (out of a maximum of 4) reflects a sufficient level, as this score reflects overweight of the response “agree” or “strongly agree” to all the items in the scale [25]. The level of eHealth literacy also differed, as scores on the eHLQ scales and between the participants differed, showing less stability than the heiQ and HLQ scales. The eHealth scores were lower in this study than in a previous study by Kayser et al [11], which can be explained by differences in age, education, and study settings. Therefore, in a rural population like the population in our study, the low level of eHealth literacy should be considered when introducing digital health services in the home care setting.

### Low eHealth Literacy

The most interesting results for health care services were the characteristics of the participants in cluster 4, with the lowest scores on the eHLQ scales, indicating low eHealth literacy knowledge (Multimedia Appendix 1). These participants were older, were mainly women, and had a lower level of education than participants in cluster 1 (with the highest level of eHealth literacy). Participants in cluster 4 seldom used a PC, laptop, tablet, or smartphone, and they seldom accessed public services generally and health care services specifically compared with participants in cluster 1. The participants in cluster 4 had several diagnoses, although not significantly more than that among participants in cluster 1. We expect their need for care and support from the home care service to be high, and this population group could benefit from digital home monitoring. Nevertheless, the results show that these participants would need more help and facilitation from the health care service than others to take advantage of digital health services. Offering nontechnological services may be a better option. Social resources that could support them in the use of digital health services must be identified if digital health services are offered. If not, they could be discriminated from receiving the same health care services as others.

### Screening for eHealth Literacy

We found that the response to the question “If someone knowing you well should assess your IT competence, would they say it is very poor/poor/average/good/very good?” was associated with several variables in the bivariate model, but only the responses to the items “use of PC, laptop, or tablet at least once a week” and “reading medical journals or test results on a national health webpage” remained significant in the multiple model (Multimedia Appendix 3). Surprisingly, no other variables remained significant in the multiple model, but the most likely explanation was the low power in our study. Further, we found a strong association between belonging to 1 of the 4 clusters and the question “If someone knowing you well should assess your IT competence, would they say it is very poor/poor/average/good/very good?” (Multimedia Appendix 1). The answer differentiated between participants in cluster 1 and those in clusters 2, 3, and 4; between participants in cluster 2 and those in cluster 3; and between participants in cluster 3 and those in cluster 4. These associations contribute to the increasing amount of data supporting the relevance and content validity of this single-item scale and indicate that a single question could be used to screen larger populations for the level of digital health skills. Those scoring low on the single question could be further assessed with multidimensional instruments such as the READHY instrument. The READHY instrument consists of 13 scales with a total of 65 items and takes 10 to 20 minutes to complete. It may be exhausting to complete, especially for old frail people with cognitive impairment. Thus, it is preferable if clinicians can scan a population and collect the same information about the technological readiness level using a single question. This single question could potentially help to increase the number of screenings to identify those in need of further investigation. Here, the READHY instrument can be used to identify specific resources and challenges in relation to the use of digitally enabling or assisting services and technologies. However, it is important to emphasize that using 1 screening item will lead to a loss in the understanding of where particular challenges are. Further, we should be aware that it only screens for digital expertise, and self-management and social support are not taken into consideration with this approach.

### Well-Being

We analyzed the variables associated with the scores of the WHO-5 (Multimedia Appendix 2). In the bivariate model, there was an association between a higher educational level and higher scores on the WHO-5, which was not significant when adjusting for other variables such as age, sex, living situation, and number of chronic diagnoses. People with a higher level of education had better health, were younger, and had better digital health literacy, which contributed to the loss of the bivariate effect of education on well-being (WHO-5) when adjusting for these variables. Further, we did not find associations of the co-habitant status and number of chronic diagnoses with well-being assessed by the WHO-5. These findings need some consideration, as other European studies have found that living alone is associated with a lower score on the WHO-5 [30-32]. One explanation may be that the context in these studies differs from that in our study (cohort living in a rural area and having long travel distances). The participants in our study were already receiving home care services and thus were in regular contact with a support person and had access to a service they could call, which may be of importance for well-being. The home care service may be equivalent to the effect of living together with others, as people receiving home care services are in regular contact with a support person and have access to a service they can call.

### Cluster Profiles

Our analysis resulted in 4 cluster profiles based on the k-means clustering analysis (Table 4). One cluster with high scores in all the READHY scales, 1 with low scores in the eHLQ scales (eHealth literacy), 1 with low scores in the heiQ scales (health education) and the HLQ scales (health literacy), and 1 with intermediate scores. Being male, being younger, having higher education, and having good access to digital devices and knowledge of IT were all associated with a better technology readiness level. This result was comparable to that in a study by Kayser et al [9], which had 1 cluster with the highest scores in the READHY scales, 1 with low scores in the eHLQ scales, and 2 involving intermediate profiles. In the Danish study by Kayser et al [9], 25% of the participants were in the cluster with the highest READHY scale scores, which is similar to the proportion of 27% in our study. When considering another study [11], comparable proportions of participants were in the group with the lowest eHLQ scale scores (13% in the previous study and 11% in our study). In another Danish study by Thorsen et al [16], it was found that 5 clusters were more meaningful for the population of patients with T2DM. They also described 1 cluster with the highest READHY scale scores and 1 with low scores on the eHLQ scales, with the 3 remaining clusters having intermediate scores [16]. In a study with Danish cancer survivors, individuals in the cluster with the highest READHY scale scores were younger than those in the other clusters [9], as in our study. On the other hand, in the study of Danish patients with T2DM, 1 of the intermediate clusters had the lowest age [16]. A recent Norwegian study recruiting 260 patients from medical and surgical wards found 6 HLQ clusters and 6 eHLQ clusters [33]. They did not collect heiQ data but found that the participants with the lowest HLQ and eHLQ scores had comparable socioeconomic clusters, similar to the participants in cluster 4 in our study [33]. Thus, the 4 profiles based on the k-means clustering analysis of our data are comparable with those in 2 Danish studies and 1 Norwegian study, despite the mean age being higher in our study.

As explained in the statistical analysis subsection in the Methods section, a confirmatory factor analysis to assess the psychometric properties of the READHY instrument was considered. However, it resulted in convergence problems, most likely due to a very small sample size with respect to the number of items. Therefore, we did not succeed in our aim to validate the Norwegian version of the READHY instrument. Instead, we reported the Cronbach α for the scales identified by a previous factor analysis of a Danish dataset [9], which showed good consistency within the assumed scales.

### Limitations

The study had several limitations. No formal power calculation was performed prior to the study, and a convenience sample was used. It is suggested that cluster analysis should only be applied when a good group separation is expected, and it is recommended to include a sample size of approximately 20 to 30 individuals per expected group [34]. Based on previous cluster analysis of the READHY instrument [9,16,33], we expected 3 to 5 clusters of individuals in our study. Thus, a sample size of 149 was sufficient according to recommendations. The inclusion of more participants in the study could strengthen our results, could allow us to perform a factor analysis, and might make it possible to report more associations from the regression models (Multimedia Appendix 2 and 3).

The data were collected by 1 to 2 health care staff in each of the 7 participating municipalities. The same research nurse trained all data collectors, and all had experience in assessing and testing the patients. However, we cannot rule out that the use of several data collectors may have caused biases in the data. On the other hand, we used well-established assessment tools that have been found to be reliable and valid. We used a long and extensive case report form (CRF) with several questionnaires. Some of the questionnaires included many questions. The participants may have become tired, especially those with impaired cognitive function, which might have influenced the data quality. The participants were given the opportunity to answer the questionnaires at their own pace and could take breaks if needed. In the end, we found that the data collected were of excellent quality, with limited missing data.

We recruited participants from only 7 municipalities in a rural region in South-Eastern Norway, with a lower education level than that generally in Norway. This fact, in addition to the fact that the participants were recruited from among those receiving home care services, may reduce the generalizability of the study. The READHY instrument should be tested in home care settings in other regions of Norway and in other countries to increase generalizability. The results from future studies and our study could be extrapolated to inform stakeholders in home care services about the need for support when introducing digital health services in their populations. The results may not be generalizable to countries without home care services or with other access to digital devices and IT. On the other hand, the participants in the study represent people who are particularly in need of digital home monitoring because of the travel distances in rural areas, and the results may be important for stakeholders and leaders of health care services in Norway and internationally. Moreover, 62 people did not consent to participate. These people were older and a larger proportion were women when compared with the cohort of participants.

### Conclusion

To our knowledge, this is the first study on eHealth literacy in people receiving home care, a population that currently is being offered and in the future will be offered digital technology. Understanding the eHealth literacy knowledge of this population is important to take advantage of digital technology. The technology readiness level varied in the cohort of Norwegian participants receiving home care, and not all people receiving home care services can be expected to benefit from digital health services. Participants in cluster 4 (n=16) had the lowest score in the eHLQ scales in the READHY instrument and should be offered nondigital services or would need extensive management support. The demographic differences between the 4 clusters may inform stakeholders about which older people need the most training and support to be able to take advantage of digital health care services. This study contributes to this important area with information about technology readiness in a population of older adults and with insights into how this is related to their perceived health and digital behaviors. We were not able to report the psychometric properties of the Norwegian version of the READHY instrument, and a larger validation study should be performed.

## Appendix

Multimedia Appendix 1 Patient profiles within the 4 clusters (N=147).

Multimedia Appendix 2 Results of linear regression analysis for the World Health Organization-Five Well-Being Index (WHO-5) (N=129).

Multimedia Appendix 3 Results of linear regression analysis for the question “IT competence assessed by others” (N=131).

## Abbreviations

eHLQ: eHealth Literacy Questionnaire
heiQ: Health Education Impact Questionnaire
HLQ: Health Literacy Questionnaire
READHY: Readiness and Enablement Index for Health Technology
T2DM: type 2 diabetes mellitus
WHO-5: World Health Organization-Five Well-Being Index
