# Supplementary material for: Assessment of Technology Readiness in Norwegian Older Adults With Long-Term Health Conditions Receiving Home Care Services: Cross-Sectional Questionnaire Study
Source: JMIR Aging. 2025 Feb 7;8:e62936. doi: 10.2196/62936 (PMC11845898; doi:10.2196/62936)
Supplement: Multimedia Appendix 1 [file aging_v8i1e62936_app1.docx]

**Multimedia Appendix 1.** Patient profiles within the 4 clusters (N=147).

| Variable | | Cluster 1 (n=40) | Cluster 2 (n=66) | Cluster 3 (n=25) | Cluster 4 (n=16) | *P* value | *P* value for pairwise comparisons, mean difference (SD), *P* value |
| --- | --- | --- | --- | --- | --- | --- | --- |
| **Age, n** | | 40 | 66 | 25 | 15 | .006^a^ | 1 vs 2: –5.1 (1.5), *P*=.001; 1 vs 4: –6.3 (2.0), *P*=.003 |
|  | Median (min, max) | 76 (61, 91) | 80 (61, 95) | 78 (64, 101) | 83 (65, 90) |  |  |
|  | Mean (SD) | 75.1 (6.5) | 80.1 (7.8) | 78.0 (9.6) | 81.4 (7.3) |  |  |
| **Sex, n** | | 40 | 66 | 25 | 16 | <.001^b^ | 1 vs 2: 41.2 (9.2), *P*<.001; 1 vs 4: 32.5 (14.0), *P*=.02; 2 vs 3: –23.2 (11.1), *P*=.04 |
|  | Women, n (%) | 12 (30.0) | 47 (71.2) | 12 (48.0) | 10 (62.5) |  |  |
| **Education, n** | | 39 | 60 | 24 | 16 | <.001^c^ | 1 vs 2: 24.4 (8.8), *P*=.01; 1 vs 3: 36.9 (10.7), *P*=.001; 1 vs 4: 41.0 (12.5), *P*=.002 |
|  | ≤12 years, n (%) | 23 (59.0) | 50 (83.3) | 23 (95.8) | 16 (100) |  |  |
|  | >12 years, n (%) | 16 (41.3) | 10 (16.7) | 1 (4.2) | 0 (0) |  |  |
| **Living situation, n** | | 40 | 66 | 25 | 16 | .08^c^ | 1 vs 2: 21.3 (9.0), *P*=.03 |
|  | Living alone, n (%) | 23 (57.5) | 52 (78.8) | 15 (60.0) | 12 (75.0) |  |  |
|  | Living with spouse/partner/other, n (%) | 17 (42.5) | 14 (21.2) | 10 (40.0) | 4 (25.0) |  |  |
| **Number of chronic diagnoses, n** | | 39 | 62 | 25 | 16 | .003^a^ | 1 vs 3: –1.2 (0.4), *P*=.003; 2 vs 3: –1.1 (0.3), *P*=.001 |
|  | Median (min, max) | 2 (0, 7) | 2 (1, 6) | 4 (1, 6) | 3 (1, 4) |  |  |
|  | Mean (SD) | 2.3 (1.5) | 2.5 (1.2) | 3.5 (1.6) | 2.8 (0.8) |  |  |
| **Use of digital devices at least once a week, n** | | 40 | 66 | 25 | 16 |  |  |
|  | PC, laptop, or tablet, n (%) | 30 (75.0) | 24 (36.4) | 16 (64.0) | 2 (12.5) | <.001^c^ | 1 vs 2: 38.6 (9.4), *P*<.001; 1 vs 4: 62.5 (12.2), *P*<.001; 2 vs 3: –27.6 (11.4), *P*=.03; 3 vs 4: 0.52 (0.14), *P*=.001 |
|  | Smartphone, n (%) | 36 (90.0) | 33 (50.0) | 13 (52.0) | 7 (43.8) | <.001^c^ | 1 vs 2: 40.0 (8.8), *P*<.001; 1 vs 3: 38.0 (10.1), *P*=.001; 1 vs 4: 46.3 (11.1), *P*=.001 |
| Use of IT in previous work or studies, n (%) | | 23 (57.5) | 11 (16.7) | 8 (32.0) | 1 (6.3) | <.001^c^ | 1 vs 2: 40.8 (8.6), *P*<.001; 1 vs 4: 51.3 (13.2), *P*=.001 |
| Use of IT to communicate with public services, n (%) | | 27 (67.5) | 11 (16.9) | 11 (44.0) | 2 (12.5) | <.001^c^ | 1 vs 2: 50.6 (8.4), *P*<.001; 1 vs 4: 55.0 (13.1), *P*<.001; 2 vs 3: –27.1 (9.8), *P*=.01; 3 vs 4: 31.5 (14.4), *P*=.045 |
| Use of a Norwegian digital identifier to access a webpage, n (%) | | 38 (95.0) | 18 (28.1) | 16 (64.0) | 3 (20.0) | <.001^c^ | 1 vs 2: 66.9 (7.7), *P*<.001; 1 vs 3: 31.0 (8.9), *P*=.002; 1 vs 4: 75.0 (8.6), *P*<.001; 2 vs 3: –35.9 (10.9), *P*=.003; 3 vs 4: 44.0 (15.1), *P*=.01 |
| Use of IT to communicate with a general practitioner, n (%) | | 20 (50.0) | 9 (13.6) | 5 (20.0) | 0 (0) | <.001^c^ | 1 vs 2: 36.4 (8.3), *P*<.001; 1 vs 3: 30.0 (12.0), *P*=.02; 1 vs 4: 50.0 (12.7), *P*<.001 |
| Logging into a national health webpage, n (%) | | 28 (70.0) | 12 (18.5) | 13 (52.2) | 1 (6.3) | <.001^c^ | 1 vs 2: 51.5 (8.4), *P*<.001; 1 vs 4: 63.8 (12.3), *P*<.001; 2 vs 3: –33.5 (10.0), *P*=.003; 3 vs 4: 45.8 (13.7), *P*=.003 |
| Use of IT to find health information on the internet or social media, n (%) | | 10 (25.0) | 2 (3.1) | 4 (16.7) | 1 (6.3) | .004^c^ | 1 vs 2: 21.9 (6.1), *P*=.001; 2 vs 3: –13.6 (5.9), *P*=.043 |
| **Reading medical journals or test results on a national health webpage, n (%)** | |  |  |  |  | <.001^c^ | 1 vs 2: ES^d^ 0.5, *P*<.001; 1 vs 4; ES 0.4, *P*=.002 |
|  | Never | 14 (35.0) | 54 (83.1) | 15 (60.0) | 14 (87.5) |  |  |
|  | Rarely | 9 (22.5) | 4 (6.2) | 4 (16.0) | 0 (0) |  |  |
|  | Sometimes | 7 (17.5) | 3 (4.6) | 3 (12.0) | 2 (12.5) |  |  |
|  | Always | 10 (25.0) | 4 (6.2) | 3 (12.0) | 0 (0) |  |  |
| **IT competence assessed by others, n (%)** | |  |  |  |  | <.001^b^ | 1 vs 2: ES^d^ 0.6, *P*<.001, 1 vs 3: ES 0.4, *P*=.009; 1 vs 4: ES 0.6, *P*<.002; 2 vs 4: ES 0.3, *P*=.01; 3 vs 4: ES 0.5, *P*=.002 |
|  | Very poor | 3 (7.5) | 16 (29.2) | 4 (16.0) | 11 (68.8) |  |  |
|  | Poor | 4 (10.0) | 28 (43.1) | 9 (36.0) | 4 (25.0) |  |  |
|  | Average | 10 (25.0) | 15 (23.1) | 8 (32.0) | 0 (0) |  |  |
|  | Good | 15 (37.5) | 2 (3.1) | 2 (8.0) | 1 (6.3) |  |  |
|  | Very good | 8 (20.0) | 1 (1.5) | 2 (8.0) | 0 (0) |  |  |
| **WHO-5^e^, n** | | 39 | 64 | 24 | 16 | <.001^a^ | 1 vs 3: 17.3 (4.6), *P*<.001; 2 vs 3: 17.7 (4.2), *P*<.001 |
|  | Median (min, max) | 64 (28, 96) | 64 (20, 100) | 48 (0, 76) | 64 (16, 80) |  |  |
|  | Mean (SD) | 63.6 (17.6) | 64.0 (17.5) | 46.3 (18.2) | 57.5 (17.6) |  |  |
| **In general, would you say your health is, n** | | 40 | 66 | 25 | 16 | <.001^c^ | 1 vs 3: ES^d^ 0.4, *P*=.005; 2 vs 3: ES 0.4, *P*=.006 |
|  | Poor | 3 (7.5) | 7 (10.6) | 10 (40.0) | 3 (18.8) |  |  |
|  | Fair | 21 (52.5) | 31 (47.0) | 12 (48.0) | 11 (68.8) |  |  |
|  | Good | 14 (35.0) | 25 (37.9) | 3 (12.0) | 2 (12.5) |  |  |
|  | Very good | 2 (5.0) | 2 (3.0) | 0 (0) | 0 (0) |  |  |
|  | Excellent | 0 (0) | 1 (5.2) | 0 (0) | 0 (0) |  |  |

^a^ANOVA.

^b^*χ^2^* test.

^c^Fisher exact test.

^d^Effect size (ES) calculated from Mann-Whitney *U* statistics is presented for ordinal variables.

^e^WHO-5: World Health Organization - Five Well-being Index.
