# Supplementary material for: Assessment of Technology Readiness in Norwegian Older Adults With Long-Term Health Conditions Receiving Home Care Services: Cross-Sectional Questionnaire Study
Source: JMIR Aging. 2025 Feb 7;8:e62936. doi: 10.2196/62936 (PMC11845898; doi:10.2196/62936)
Supplement: Multimedia Appendix 3 [file aging_v8i1e62936_app3.docx]

**Multimedia Appendix 3.** Results of linear regression analysis for the question “IT competence assessed by others” (N=131).

| Variable | Bivariate models | | Multiple model | |
| --- | --- | --- | --- | --- |
|  | RC (95% CI) | *P*-value | RC (95% CI) | *P*-value |
| Age  Sex, man  Education, >12 years  Living situation, Living with spouse/partner/other  Chronic diagnosis, number  Use of PC/laptop/tablet at least once a week  Use of smartphone at least once a week  IT used to communicate with general practitioner  Used IT to find health information at the internet or social media  Read your medical journal or test results on national health webpage sometimes/always  In general, would you say your health is.. | -0.05 (-0.07; -0.02)  0.73 (0.31; 1.16)  1.14 (0.63; 1.65)  0.42 (-0.01; 0.86)  0.06 (-0.10; 0.23)  1.57 (1.24; 1.90)  1.19 (0.84; 1.55)  1.49 (1.08; 1.91)  1.57 (1.11; 2.04)  1.61 (1.21; 2.01)  0.11 (-0.19; 0.41) | **<.001**  **.001**  **<.001**  .057  .445  **<.001**  **<.001**  **<.001**  **<.001**  **<.001**  .465 | -0.02 (-0.04; 0.001)  0.26 (-0.03; 0.56)  0.22 (-0.20; 0.64)  -0.24 (-0.57; 0.09)  0.04 (-0.07; 0.14)  0.98 (0.61; 1.34)  0.21 (-0.13; 0.54)  0.29 (-0.15; 0.74)  0.30 (-0.22; 0.83)  0.84 (0.41; 1.27)  0.005 (-0.19; 0.20) | .060  .075  .302  .159  .492  **<.001**  .221  .194  .256  **<.001**  .956 |

IT=Information Technology; RC=Regression Coefficient, CI=Confidence Interval, bold=*P*<.05
